# The *Spot the Troll Quiz* game increases accuracy in discerning between real and inauthentic social media accounts

**DOI:** 10.1093/pnasnexus/pgad094

**Published:** 2023-03-22

**Authors:** Jeffrey Lees, John A Banas, Darren Linvill, Patrick C Meirick, Patrick Warren

**Affiliations:** John E. Walker Department of Economics, Clemson University, Clemson, SC 29634, USA; Media Forensics Hub, Clemson University, Clemson, SC 29634, USA; Andlinger Center for Energy and the Environment, Princeton University, Princeton, NJ 08540, USA; Department of Communication, University of Oklahoma, Norman, OK 73019, USA; Media Forensics Hub, Clemson University, Clemson, SC 29634, USA; Department of Communication, Clemson University, Clemson, SC 29634, USA; Department of Communication, University of Oklahoma, Norman, OK 73019, USA; John E. Walker Department of Economics, Clemson University, Clemson, SC 29634, USA; Media Forensics Hub, Clemson University, Clemson, SC 29634, USA

**Keywords:** disinformation, misinformation, social media, Twitter, trolls, inoculation

## Abstract

The proliferation of political mis/disinformation on social media has led many scholars to embrace “inoculation” techniques, where individuals are trained to identify the signs of low-veracity information prior to exposure. Coordinated information operations frequently spread mis/disinformation through inauthentic or “troll” accounts that appear to be trustworthy members to the targeted polity, as in Russia's attempts to influence the 2016 US presidential election. We experimentally tested the efficacy of inoculation against inauthentic online actors, using the *Spot the Troll Quiz*, a free, online educational tool that teaches how to spot markers of inauthenticity. Inoculation works in this setting. Across an online US nationally representative sample (*N* = 2,847), which also oversampled older adults, we find that taking the *Spot the Troll Quiz* (vs. playing a simple game) significantly increases participants’ accuracy in identifying trolls among a set of Twitter accounts that are novel to participants. This inoculation also reduces participants’ self-efficacy in identifying inauthentic accounts and reduced the perceived reliability of fake news headlines, although it had no effect on affective polarization. And while accuracy in the novel troll-spotting task is negatively associated with age and Republican party identification, the Quiz is equally effective on older adults and Republicans as it was on younger adults and Democrats. In the field, a convenience set of Twitter users who posted their *Spot the Troll Quiz* results in the fall of 2020 (*N* = 505) reduced their rate of retweeting in the period after the *Quiz*, with no impact on original tweeting.

**Significance Statement**

Misinformation is frequently spread by inauthentic social media accounts. Here, we test the effectiveness of the *Spot the Troll Quiz*, a free, online educational tool designed to teach individuals to discern between real and “troll” accounts. In a large, online experiment, we find that inoculation through taking the *Spot the Troll Quiz* significantly improves one's ability to accurately identify accounts used by foreign governments to influence political discourse in the United States. Importantly, the benefits of the *Spot the Troll Quiz* were consistent for those across the political spectrum and across all ages. In a smaller convenience field sample, taking the Quiz seems to reduce retweeting without impacting original tweets.

## Introduction

Misinformation is difficult to debunk (1–3,), leading researchers to focus on preventative approaches like “prebunking,” based on inoculation theory (4). Inoculation theory posits that inducing resistance to persuasion is analogous to viral vaccination. For example, attitudinal inoculation introduces and refutes arguments counter to one's current position, prompting a range of resistance–promotion actions that facilitate resistance to subsequent persuasive messaging (4–6). Inoculation interventions aimed at online misinformation incorporate critical thinking.

Critical thinking intervention studies have revealed promising results in the fight against online misinformation. Lutzke et al. (7) found that critical thinking interventions about evaluating online information reduced the likelihood to trust, like, and share climate change misinformation, but did not diminish perceptions of legitimate information. Guess et al. (8) found that seeing Facebook's “Tips to Spot Fake News” improved recognition of fake news in the United Sates and India. A nudge campaign merely reminding people to think about accuracy when judging a headline on social media increased their discernment in their willingness to share true vs. false headlines (9). Although past interventions have addressed the perceived reliability of accounts impersonating companies (10), one noteworthy distinction of the work presented here is the novel focus on distinguishing between real-world inauthentic and authentic online accounts.

Inoculation interventions aimed at combating misinformation campaigns have incorporated elements of critical thinking. Inoculation has been implemented across a wide range of topics [see (11) for meta-analysis] and in the context of misinformation has been proposed as an alternative to fact-checking interventions (12, 13). Inoculation research has traditionally been limited by the ability to predict persuasive appeals; in order to inoculate against misinformation campaigns, scholars have typically needed to anticipate specific claims, topics, and/or arguments and then preemptively refute them. To overcome these challenges, researchers have created interventions that reveal the influence tactics used in disinformation campaigns. For example, scholars utilized an Internet-based game, *Bad News*, to inoculate against the underlying tactics of disinformation instead of specific claims (10, 14, 15). *Bad News* builds on previous research aimed at teaching the faulty logic behind conspiracy theories (16, 17), as well as using inoculation to expose deceptive argumentation tactics (18). Compared with controls, *Bad News* consistently reduced the perceived reliability of fake news headlines, and its effectiveness has replicated across multiple countries and lasts over 3 months when played regularly (19). Similar effects have been found for games designed to inoculate against manipulation techniques related to the COVID-19 (20), elections (21), and climate change (22).

Building on previous inoculation interventions, our project examines the influence of the *Spot the Troll Quiz*. The Quiz is a gamified inoculation intervention. Unlike previous interventions, the *Spot the Troll Quiz* is aimed at teaching individuals to identify fake online profiles created by real-world actors, instead of fake content or fake accounts created by researchers (10, 15). It teaches individuals to spot tactics commonly used in deception and persuasion, in general (23, 24), and online inauthenticity such as phishing (25, 26) and disinformation (27) specifically, including the presence of extreme and hyperbolic content; the use of young, attractive, and often female profile images; spreading of hoax events; the conspicuous lack of personal information; claiming identification with affinity groups with no identifiable members; and exclusive focus on prominent individuals and national news. The Quiz's focus on *general* tactics of deception, taught with clear examples of accounts created by the Russian Internet Agency juxtaposed to the behavior of authentic users, allows Quiz takers to generalize what they learn beyond the narrow context of the 2016 Russian disinformation campaigns. Another key difference between the *Spot the Troll Quiz* and similar interventions is that while gamified interventions often increase people's confidence in their ability to identify misinformation (20, 21), this intervention was meant to make people aware of the difficulty of spotting a troll, i.e. an inauthentic account run by foreign coordinated information operations attempting to influence users. Those low in ability are likely to be overconfident in their ability (28, 29), and overconfidence in one's news judgments is associated with greater exposure to fake news and less accurate discernment between true and false topical claims (30). We expected (and found) confidence to be lower among those who took *Spot the Troll Quiz*, even as actual ability increased.

**Fig. 1.**
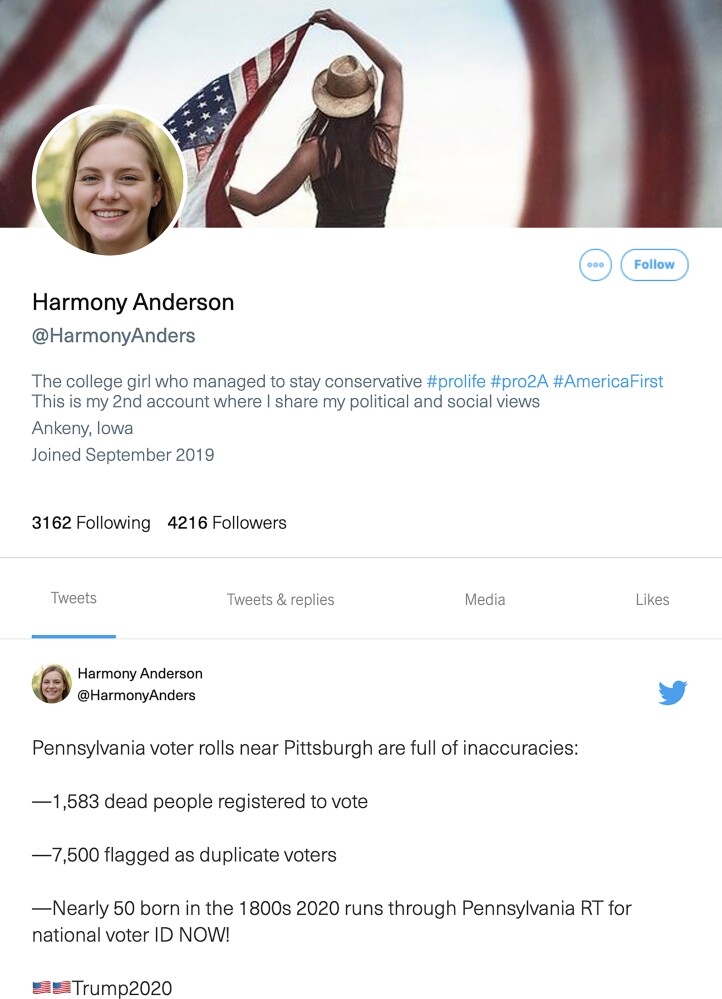
Image of the top section of a profile (one of eight) that Quiz takers are asked to read and then guess whether the profile is real or a troll account. Users could scroll down to see several more tweets not visible in Fig. 1.

**Fig. 2.**
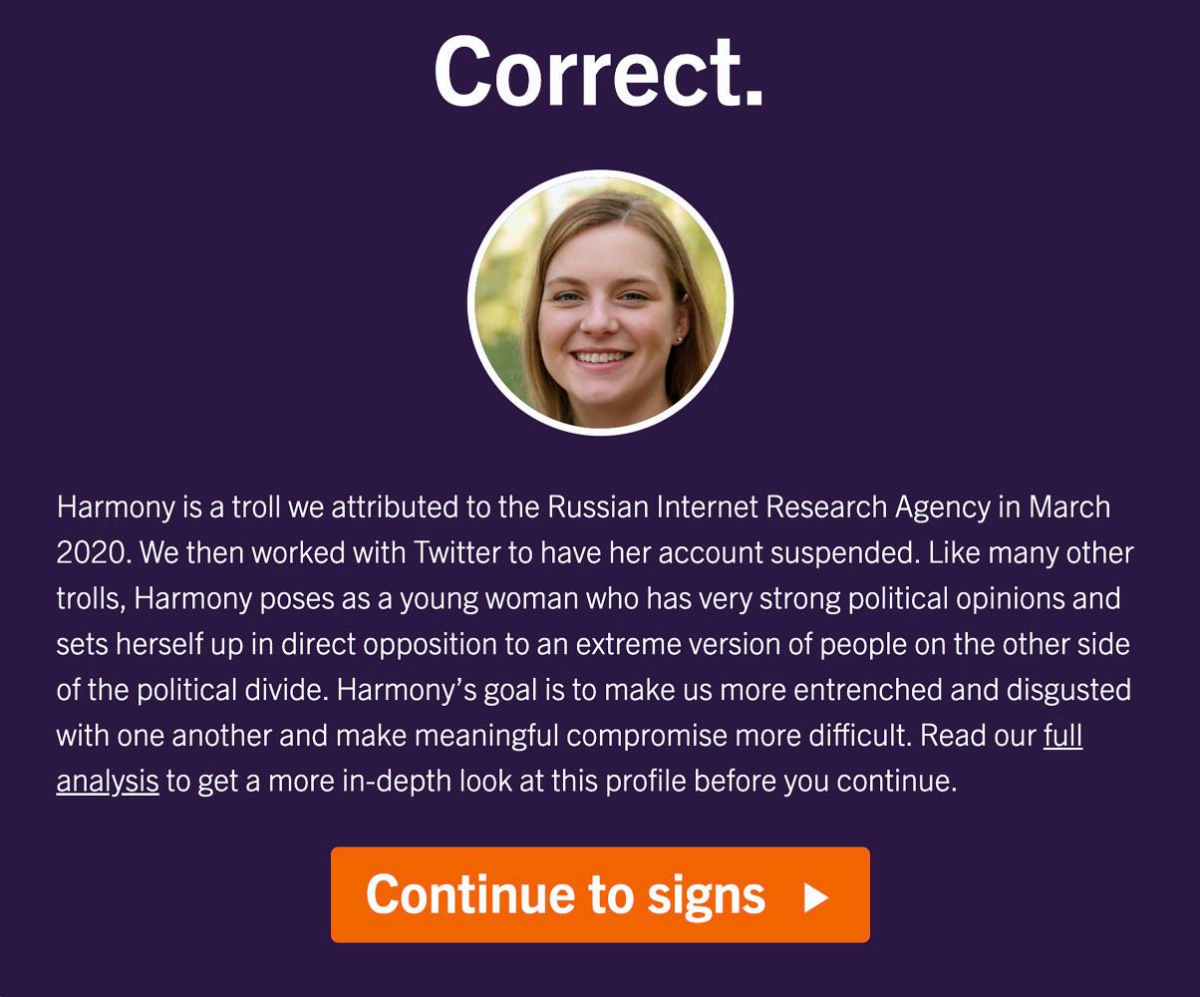
The message Quiz takers get after (correctly) identifying this profile as a troll. The subsequent “signs” page provides Quiz takers with detailed visual information regarding what in this profile served as evidence it was a troll account.

The *Spot the Troll Quiz* is an inoculation intervention created by the Media Forensics Hub at Clemson University. It was created by an academic team who has worked with various platforms to identify and remove accounts operated by foreign information operation campaigns and as such has deep personal and scientific knowledge of the nature of such troll accounts. The tool has gamified elements as gamification has been found to contribute to effective instruction (31) and has been used by past disinformation interventions (15). Its goal is to get users to be more considerate regarding who they engage with and to identify the markers of inauthenticity (32). Designed to teach users tactics employed by disinformation operations, the Quiz is a series of questions which build off one another in a scaffolded manner. Each question presents a social media profile (see Fig. 1), some of which are genuine and others of which were created by the Russian Internet Research Agency. The user decides which are authentic and which are not, receiving tips and context following each profile (see Fig. 2). Whether or not Quiz takers correctly identify each account, after their guess, they are given detailed information about what attributes of the account signal whether it was authentic or inauthentic. This ensures that all users, regardless of their performance, receive the same information. To date, the Quiz has been accessed more than 1 million times and used around the world by both educators and individual users.

We conducted two studies to examine the effects of the *Spot the Troll Quiz*. The first was an experimental study where participants across two samples (a nationally representative quota sample and a convenience sample of people aged 60 and older) were randomly assigned to take the Quiz or play a video game (control condition) and then administered a novel troll-spotting task, where participants were asked to identify whether eight true accounts (even balance of real/troll accounts and liberal/conservative accounts) were real people or trolls, along with other dependent variables. The troll accounts used in the novel troll-spotting task were taken from Twitter releases of information operations conducted by Russia, Iran, and Venezuela. The second was an observational study where we observed the naturalistic behavior of Twitter users in the days after they publicly posted their *Spot the Troll Quiz* scores. The experimental study and hypotheses were preregistered (https://osf.io/jyxzq/). We hypothesized that taking the Quiz (relative to control) would [Hypothesis 1 (H1)] reduce social media inauthenticity self-efficacy, as the Quiz clearly demonstrates to participants the ways in which their judgments were wrong; [Hypothesis 2 (H2)] reduce affective polarization, as the Quiz makes clear that trolls attempt to make us more politically polarized and want to make political compromise more difficult and impersonate both liberal and conservative Americans; [Hypothesis 3 (H3)] increase the accuracy of identifying novel troll and real social media accounts, as the Quiz provides clear information about the attributes of fake and real accounts; and [Hypothesis 4 (H4)] increase/decrease the perceived reliability of real/fake news headlines, as the inoculation treatment and lessons of the Quiz may also offer some cross-protection against inauthentic information, a related concern (33).

## Results

### Experimental study

Results below are reported for analyses conducted on the combined sample (*N* = 2,847): a single data set including the representative quota sample and a sample of people ages 60 and older. Analyzing the combined sample was preregistered, and in general, results are consistent across both subsamples. Age effects are described in the subsequent section, and full results and regression tables for all analyses across both subsamples can be found in the online Supplementary material. Unless stated, all analyses below control for party identification (as preregistered). All analyses below are also robust to controlling for education, ethnicity, age, and gender (which was not preregistered, see online Supplementary material for results with demographic controls). Unless stated, all regression coefficients represented with a *β* are standardized (*z*-scored continuous variables) and can be interpreted as changes in units of Sd. The *b* coefficients reported in the Observational study are unstandardized as they represent count data.

#### Combined sample results

In support of H1, the *Spot the Troll Quiz* caused a reduction in social media inauthenticity self-efficacy. Participants who took the Quiz reported significantly lower social media inauthenticity self-efficacy [mean (*M*) = 3.91, Sd = 1.16] than participants in the control condition (*M* = 4.50, Sd = 1.10) [*β* = −0.51 (−0.58, −0.43), *t*(2,843) = −13.93, *P* < 0.001]. The effect of the condition on social media inauthenticity self-efficacy did not differ by subsample [*F*(1, 2,841) = 0.13, *P* = 0.717].

Contrary to H2, the *Spot the Troll Quiz* did not significantly reduce affective polarization, operationalized as the difference between in-party and out-party liking, among Democrats and Republicans (True Independents not analyzed). There was no difference in affective polarization between the Quiz (*M* = 51.5, Sd = 32.7) and control (*M* = 50.6, Sd = 33.7) conditions [*β* = 0.03 (−0.02, 0.11), *t*(2,579) = 0.68, *P* = 0.497]. The same pattern emerged when operationalizing affective polarization as only out-party liking (see online Supplementary material). The effect of the condition on polarization did not differ by subsample [*F*(1, 2,577) = 0.34, *P* = 0.559], and polarization was uncorrelated with all other dependent variables (see Table S1).

In support of H3, the *Spot the Troll Quiz* caused a significant increase in the accuracy of responses to the novel troll-spotting task, where participants attempted to accurately identify whether eight true Twitter accounts (four real people, four troll, even split of liberal/conservative) were real people or trolls. Accuracy was operationalized as the sum of correct responses across the eight profiles. Participants in the Quiz condition were significantly more accurate (*M* = 4.25, Sd = 1.44) relative to the control condition (*M* = 3.82, Sd = 1.58) [*β* = 0.29 (0.21, 0.36), *t*(2,843) = 7.77, *P* < 0.001]. The effect of the condition on accuracy did not differ by subsample [*F*(1, 2,841) = 0.34, *P* = 0.559]. Republicans were consistently less accurate (*M* = 3.87, Sd = 1.56) than Democrats (*M* = 4.24, Sd = 1.49) [*β* = −0.24 (−0.32, −0.17), *t*(2,843) = −6.33, *P* < 0.001]. However, party identification did not significantly interact with the effect of the condition on accuracy [*F*(2, 2841) = 2.65, *P* = 0.071], suggesting that while Republicans were less accurate on average, the effect of the *Spot the Troll Quiz* on the novel troll-spotting task was consistent across all partisans.

The effect of the Quiz on accuracy in the novel troll-spotting task varied by the nature of the accounts. To examine this, we reperformed the models above, but shifted the outcome to either the sum of correct responses to the four troll accounts or four real accounts. The Quiz caused a significant increase in the accuracy of identifying the four troll accounts (*M* = 2.62, Sd = 1.03) relative to control (*M* = 1.97, Sd = 1.04) [*β* = 0.62 (0.55, 0.69), *t*(2,843) = 17.50, *P* < 0.001]. However, the Quiz caused a significant decrease in the accuracy of identifying the four real accounts (*M* = 1.61, Sd = 0.94) relative to control (*M* = 1.85, Sd = 1.04) [*β* = −0.24 (−0.31, −0.17), *t*(2,843) = −6.45, *P* < 0.001]. These results suggest that while the Quiz did increase skepticism toward the authenticity of all accounts viewed, skepticism was not blind. Interestingly, despite significant lower self-efficacy after taking the Quiz (H1, above), after completing the novel troll-spotting task, participants in the Quiz condition reported higher estimates of their own accuracy (*M* = 4.42, Sd = 1.68) relative to those in the control condition (*M* = 3.99, Sd = 1.88) [*β* = 0.24 (0.17, 0.32), *t*(2,843) = 6.50, *P* < 0.001], suggesting that they felt that the Quiz helped their performance on the task.

To test the robustness of the effect of the Quiz on identifying false and real accounts, we performed two nonpreregistered analyses. The first was of *troll discernment* (akin to “fake news discernment”; (34), operationalized as participant's accuracy in identifying a troll account (0 to 4) minus the extent to which they improperly marked a real account as a troll (0 to 4). Across the sample, discernment was significantly greater than zero (*M* = 0.58, Sd = 1.34) [*t*(2,846) = 22.91, *P* < 0.001], suggesting participants on average were able to discern troll accounts from real accounts, and discernment was higher in the Quiz condition (*M* = 0.67, Sd = 1.34) than that in the control condition (*M* = 0.47, Sd = 1.34) [*β* = 0.15 (0.07, 0.22), *t*(2,843) = 3.99, *P* < 0.001]. The effect of the condition on discernment did not differ by subsample [*F*(1, 2,841) = 0.01, *P* = 0.906]. Party identification significantly interacted with the effect of the condition on discernment [*F*(2, 2841) = 3.45, *P* = 0.032]. Marginal mean estimation (see Table 1 for discernment and accuracy means) found that Democrats had higher discernment than Republicans [*β* = 0.31 (0.22, 0.40), *t*(2,841) = 8.01, *P* < 0.001] and True Independents [*β* = 0.22 (0.06, 0.37), *t*(2,841) = 3.25, *P* = 0.003], and the Quiz caused a significant increase in discernment among Republicans [*β* = 0.22 (0.11, 0.33), *t*(2,841) = 4.00, *P* < 0.001] and True Independents [*β* = 0.30 (0.06, 0.54), *t*(2,841) = 2.47, *P* = 0.014], but not Democrats [*β* = 0.04 (−0.06, 0.15), *t*(2,841) = 0.81, *P* = 0.416]. These findings suggest that the Quiz was most helpful on the participants who were least discerning at baseline.

**Table 1.** Marginal means and Sd of troll discernment and accuracy by condition and party identification.

Condition	Party ID	Discernment	Sd_dis_	Accuracy	Sd_acc_
Control	Democrat	0.76	1.31	4.09	1.56
Control	Republican	0.22	1.33	3.59	1.59
Control	True Independent	0.29	1.26	3.56	1.39
Quiz	Democrat	0.82	1.28	4.39	1.40
Quiz	Republican	0.52	1.38	4.13	1.47
Quiz	True Independent	0.70	1.38	4.17	1.42

As a second robustness test, we retested H3 using linear mixture models instead of averaging accuracy across stimuli within participants, as suggested by Pennycook et al. (35). Accuracy in judging each of the eight accounts was modeled with crossed random intercepts for participant and for account, and fixed effects for condition and party identification. We replicated the result that the Quiz caused a significant increase in accuracy [*β* = 0.11 (0.08, 0.14), *t*(2,843) = 7.77, *P* < 0.001] and that Republicans were less accurate than Democrats [*β* = −0.09 (−0.12, −0.06), *t*(2,843) = −6.33, *P* < 0.001].

**Fig. 3.**
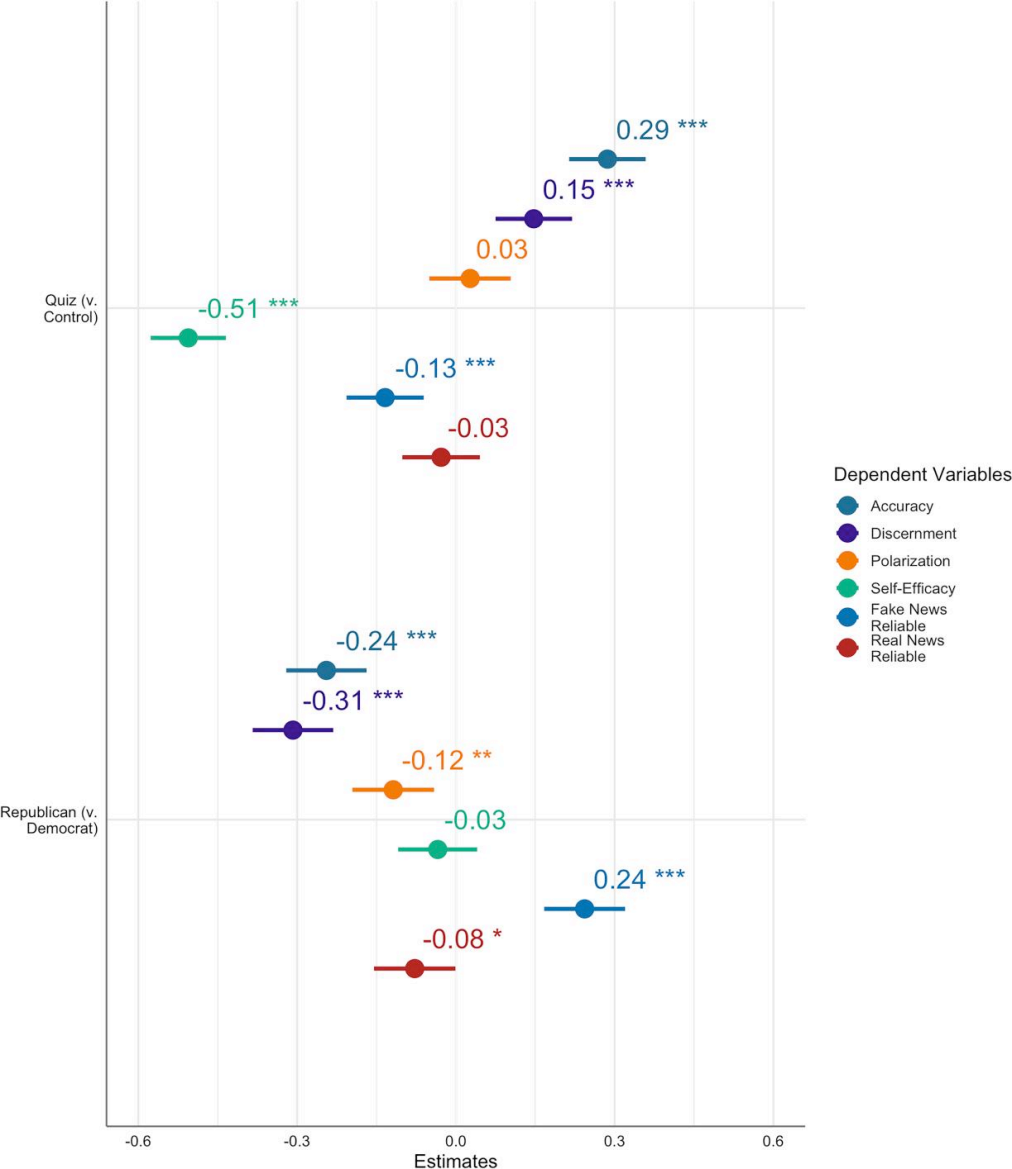
Plot of standardized OLS regression estimates from the models testing the stated hypotheses. The top row displays the condition effect of Quiz vs. control on the dependent variables, and the bottom row displays the fixed effect partisan difference for Republicans vs. Democrats in the models testing for condition effects. Bars at 95% CI. *P < 0.05, **P < 0.01, and ***P < 0.001.

In partial support for H4, participants rated fake news headlines as less reliable in the Quiz condition (*M* = 3.24, Sd = 1.12) compared with the control condition (*M* = 3.39, Sd = 1.11) [*β* = −0.13 (−0.21, −0.06), *t*(2,842) = −3.60, *P* < 0.001], but did not rate real news as more reliable in the Quiz condition (*M* = 4.92, Sd = 1.17) compared with the control condition (*M* = 4.95, Sd = 1.18) [*β* = −0.03 (−0.10, 0.05), *t*(2,842) = −0.75, *P* = 0.451]. Republicans rated fake news headlines are more reliable than did Democrats [*β* = 0.24 (0.17, 0.32), *t*(2,842) = 6.24, *P* < 0.001] and real news as less reliable than did Democrats [*β* = −0.08 −(0.15, −0.001), *t*(2,842) = −1.99, *P* = 0.047]. See Fig. 3 for the estimate plot of the Quiz and partisan effects.

#### Age effects

Age was negatively associated with social media inauthenticity self-efficacy, controlling for the condition [*β* = −0.23 (−0.26, −0.19), *t*(2,842) = −12.99, *P* < 0.001], and this association was not moderated by the condition [*F*(1, 2,841) = 0.87, *P* = 0.352]. Age was positively associated with affective polarization, controlling for the condition [*β* = 0.24 (0.20, 0.28), *t*(2,577) = 12.52, *P* < 0.001], and this association was not moderated by the condition [*F*(1, 2,577) = 1.24, *P* = 0.265]. Age was not associated with either the perceived reliability of fake news headlines [*β* = 0.00 (−0.03, 0.04), *t*(2,840) = 0.16, *P* = 0.872] or the perceived reliability of real news [*β* = −0.02 (−0.06, 0.02), *t*(2,840) = −1.12, *P* = 0.263].

Age was negatively associated with accuracy on the novel troll-spotting task in the combined sample, controlling for the condition [*β* = −0.15 (−0.12, −0.11), *t*(2,840) = −6.60, *P* < 0.001]; however, this relationship was qualified by a significant interaction with party identification [*F*(2, 2840) = 6.80, *P* < 0.001]. Simple slope analysis revealed that the slope between age and accuracy was significantly larger for Republicans [*β* = −0.26 (−0.32, −0.20)] than Democrats [*β* = −0.12 (−0.17, −0.08)] [*β*_difference_ = 0.13, *t*(2,840) = 3.45, *P* = 0.002]. There was no significant interaction between age and condition in predicting accuracy [*F*(1, 2,839) = 0.93, *P* = 0.661], and the effect size of the Quiz on accuracy (vs. control) is unchanged when age is included in the model (both *β* = 0.29). Additionally, the association between age and accuracy was robust in controlling for whether participants reported being regular (at least once a month) Twitter users [*β* = −0.16 (−0.20, −0.12), *t*(2,841) = −8.59, *P* < 0.001]. Due to concerns over possible nonlinear interaction effects (36), we transformed age into a five-group categorical variable and interacted it with the condition. As with the linear analysis, older individuals were uniformly less accurate, and no significant interaction of age and condition was observed [*F*(4, 2835) = 0.52, *P* = 0.719]. These results overall suggest that older adults were less able to accurately identify troll and real accounts compared with younger adults, but that the effectiveness of the Quiz was equally strong for participants of all ages.

### Observational study

The observational study consisted of measuring the organic Twitter behavior of a convenience sample of the 505 people who voluntarily posted a score for the *Spot the Troll Quiz* on their publicly available Twitter feed, using the automated “Share Your Score” options, between 2020 September 15 and 2020 October 30. For each poster, we observed all tweets they posted between 2020 August 19 and 2020 November 14 that still existed on their account as of 2020 November 14, the date of collection. This record included both original tweets and retweets, for a total of 245,962 tweets. In the case of retweets, we also used information about the accounts they retweeted, both on the date of collection and again on 2022 July 17.

For each account, we defined the event day as the day on which they posted their score on the *Troll Quiz*. We built a 3-week window before and after the treatment day and performed a panel event–study design to contrast behavior before and after that day, adjusting for the general behavior in the overall population on each calendar day with fixed effects for calendar day. This design will accurately estimate the impact of the *Quiz* as long as no other factors that drive posting behavior consistently co-occur with Quiz taking/posting.

**Fig. 4.**
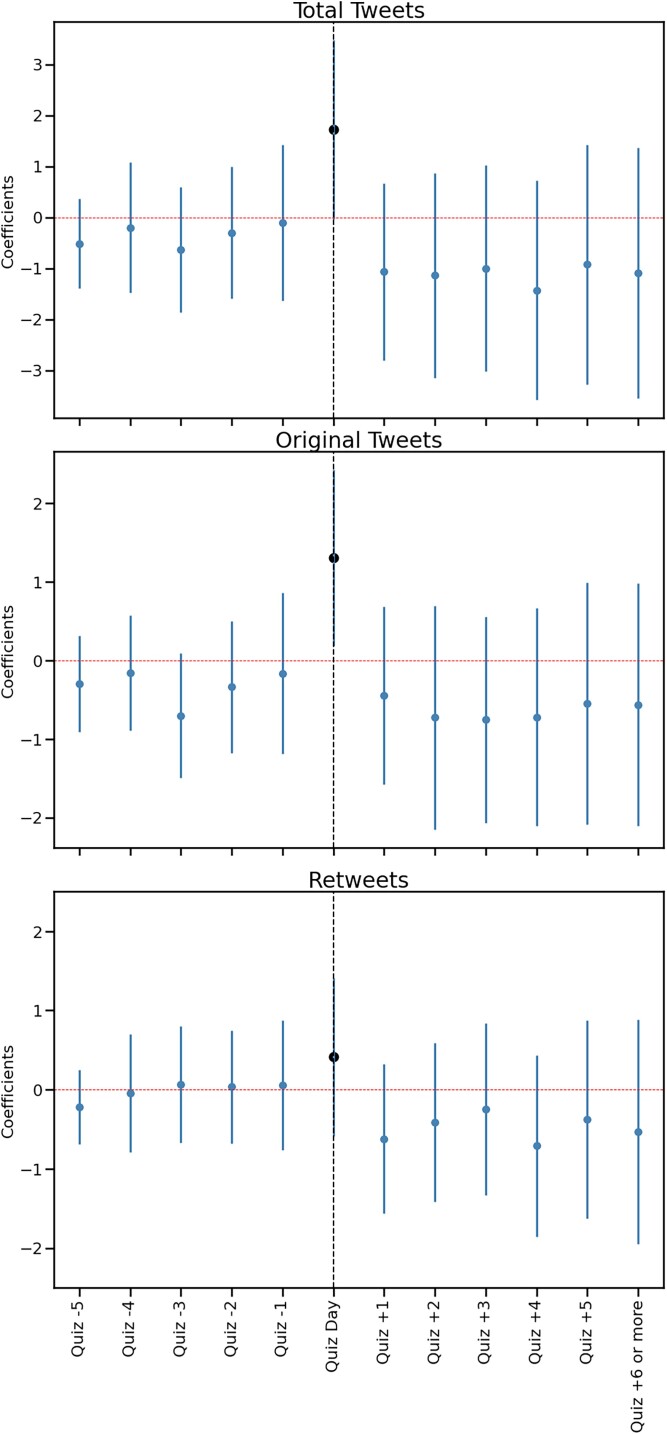
Total tweets, original tweets, and retweets per day, relative to 6+ days before Quiz. Coefficients and 95% CI from three separate panel regressions including day and account fixed effects.

We found significant changes in several dimensions of behavior in the days after taking the Quiz. Treated individuals reduced their rate of tweeting [*b* = −1.69 (−2.89, −0.492), *t*(19,036) = −2.77, *P* = 0.006], a decline of about 19% in tweets per day (*M* = 8.98, Sd = 16.6). This consequence was most apparent in the context of retweets [*b* = −0.97 (−1.63, −0.310), *t*(19,036) = −2.88, *P* = 0.004], a decline by about 26% in retweets/day (*M* = 3.71, Sd = 9.55). There was no statistically significant effect on original tweeting, although the point estimates are small and negative [*b* = −0.72 (−1.46, 0.031), *t*(19,036) = −1.88, *P* = 0.060], a decline by about 14% in original tweets/day (*M* = 5.27, Sd = 10.6). Figure 4 presents estimated coefficients for a day-by-day version of this regression, with all days more than 6 days before the Quiz as the excluded (reference) period. There was no evidence of a pretrend in the coefficients. The spike in posting at day 0 indicates high levels of activity on the Quiz-share day. The behavior on all days more than 5 days after the Quiz is pooled into the final bucket. These data suggest that taking the Quiz was associated with a decrease in sharing behaviors on Twitter and that decline persists throughout the posttest period.

In July 2022, we also revisited the accounts that were retweeted to see if they were no longer on Twitter, either because they deleted their account or because they were suspended by Twitter. Retrospectively, the sharing of retweeted accounts which are no longer on Twitter serves as a signal, albeit a noisy signal, of sharing low-quality information, as accounts which have been suspended are plausibly more likely sources of low-quality information relative to the median Twitter user. We found that an average of 0.148 tweets per day were retweets from accounts that are no longer on Twitter. In the days after taking the Quiz, retweeting of subsequently deleted accounts did not change [*b* = −0.018 (−0.059, 0.024), *t*(16,987) = −0.825, *P* = 0.409]. Thus, we find no evidence that Quiz taking is particularly correlated with a reduction in sharing one broad class of potentially low-quality accounts.

## Discussion

The purpose of the experiment was to investigate the effects of a critical thinking inoculation intervention, the *Spot the Troll Quiz*, on the accuracy of perceptions during a novel troll-spotting task and related perceptions, in both a nationally representative and older sample. Participants who took the Quiz reported significantly less confidence in their ability to identify false information online; however, those who took the Quiz significantly outperformed the control participants on accurately identifying Twitter profiles’ authenticity. This increased accuracy in the novel troll-spotting task was largely driven by accuracy in identifying fake accounts and partially offset by lower accuracy in identifying real profiles. However, when accuracy was operationalized as the ability to discern troll accounts from real accounts (rather than raw accuracy), the Quiz too significantly increased participants’ discernment. Participants who took the Quiz also correctly believed they performed better on the novel troll-spotting task relative to participants in the control condition. Additionally, the Quiz caused participants to perceive a series of fake news headlines as less reliable sources of information, although the Quiz did not cause a reduction in affective polarization.

In a separate observational study of the Twitter behavior of a convenience sample who took the Quiz, we found they were subsequently less likely to post retweets compared with a control set of Twitter users, with no change in their posting of original tweets. This pattern in the observational study is consistent with the experimental finding that the Quiz reduces takers’ confidence in their ability to spot inauthentic accounts, which in turn leads to a reticence to share content from others. The persistence of this reduction in retweeting behavior suggests the reduction in confidence persists for many days. It is difficult to measure a change in discernment in the observational data, since we lack reliable labels for inauthentic accounts and inauthentic accounts are, in fact, quite rare. Our rough attempt to proxy with accounts that are now suspended or deleted revealed no strong evidence for impacts on discernment, in practice.

The present research contributes to the literature on misinformation by focusing on a novel problem, the identification of deceptive social media accounts, rather than the identification of false information/headlines. Our data demonstrate the efficacy of the *Spot the Troll Quiz* for not only improving the ability to recognize inauthentic online accounts, but also inhibiting susceptibility to believing fake news headlines. This free education tool, which is based on actual troll profile data from social media companies, utilizes an interactive, gamified format and demonstrates clear results on both attitudes and behaviors. The scalability issue that has hampered previous inoculation interventions [see (3)] is not an issue with the *Spot the Troll Quiz*, and our data contribute to the growing literature of gamified interventions that incorporate active user participation to maximize resistance to misinformation uptake (14, 21, 37). The driving force behind the results appears to be an increased awareness of, and accompanying decrease in confidence to spot, inauthentic content and profiles on social media. The experiment demonstrated a decrease in social media inauthenticity self-efficacy among Quiz takers as well as an overestimation of fake profiles among the real profiles (though an overall increase in discernment), which is consistent with the observational study's findings that the Quiz reduced retweeting overall. Given the overall overestimation of the ability to spot instances of misinformation (30), or even deception generally, perhaps a reduction in overconfidence is warranted. Further, the Quiz only teaches about troll profiles, but the increased awareness of online deception transferred to increased suspicion of fake news headlines, demonstrating the umbrella inoculation effect.

Another contribution of this study is its focus on older adults. Studies show older adults spread more misinformation online than younger people (38–40), so there is considerable practical importance in studying interventions designed to induce resistance to misinformation online. In addition to a nationally representative sample, we collected an additional sample of 937 people aged 60 and above in order to systematically examine how age interacts with our inoculation intervention. Despite the experimental results replicating across political identification, age was negatively correlated with accuracy in the novel troll-spotting task. Age interacted with political affiliation in that older Republicans were the least accurate at assessing profile veracity. Future research should continue to study how to help older adults identify misinformation.

Future research should also further explore the implication of our finding that the intervention increased participant skepticism toward the authenticity of all social media accounts. This finding, which is consistent with past media literacy interventions (8), has potential social benefits; general skepticism may be a strong defense against being fooled by inauthentic actors. Some academics, however, have cautioned that too much skepticism may be a poor result of such interventions as users are left not knowing what to trust (41). Where the correct balance lies requires further inquiry.

As with all studies, there are limitations to the present study that merit discussion. The cross-sectional nature of the experimental data is a limitation, as a snapshot precludes the analysis of the long-term effects of the Quiz on misinformation-related outcomes. Similarly, the observational study involves the behaviors of individuals who chose to post their Quiz scores publicly, making broader inferences to general behaviors on Twitter limited. Future research should examine how the process of critical thinking interventions unfolds over time. Relatedly, the troll behaviors identified by the Quiz and included in the novel troll-spotting task are specific to a time frame, namely, within a year of the 2016 US presidential election, and therefore, external validity may be limited as future trolls develop new tactics. Another limitation is the low statistical power to detect partisan interactions with condition effects. Strong inferences to the null here should be avoided, and future research ought to explore the one significant interaction we did observe, namely, that the Quiz increased discernment for Republicans and Independents but not Democrats. Lastly, the use of real-world social media accounts, both real troll and authentic users across the Quiz and the novel troll-spotting task, meant we sacrificed the ability to examine mechanistic questions (e.g. to control for and/or measure whether participants attend to the presence of personally identifying information in the profiles) in order to obtain greater accessibility in the Quiz and generalizability in the results. Future research would benefit from tighter controls over stimuli in order to test mechanistic questions regarding what causes participants to be more accurate in the identification of inauthentic accounts.

While past research on combating mis/disinformation has understandably focused on inoculation and nudge interventions to reduce the belief in and sharing of fake news, the *Spot the Troll Quiz* demonstrates the utility of inoculation interventions in a novel domain: the identification of inauthentic social media account associated with State-sponsored coordinated information operations. The *Spot the Troll Quiz* is an effective, free, accessible, and scalable resource for educators and scholars alike hoping to assist the public in identifying and avoiding inauthentic online actors and the misinformation they spread.

## Materials and methods

### Experimental study

#### Ethics statement

This study was approved by the University of Oklahoma Institutional Review Board. All participants gave informed consent to participate. No deception was utilized.

#### Open science and preregistration

This study's design, hypotheses, and analyses were preregistered (https://osf.io/jyxzq). All study materials, anonymized data, and analysis code are publicly available (https://osf.io/kwmqv/).

#### Samples

Participants were recruited for two simultaneous samples: a US nationally representative sample and a convenience sample of US Americans age 60+, both recruited through Forthright Access survey panels (https://www.forthrightaccess.com/) during April–May 2022. The US nationally representative sample was a nonprobability sample quota-matched to census distributions of age, gender, region of the United States, ethnicity, and political party identification. The 60+ sample was a convenience sample of individuals who identified as being at least 60 years of age. We targeted 2,000 participants in the representative sample and 1,000 participants in the 60+ sample. A total of 282 participants (164 in the representative sample and 118 in the 60+ sample) failed an overt attention check at the beginning and were prevented from completing the survey (per preregistration), leading us to collect 2,042 in the representative sample and 1,005 in the 60+ sample who completed the survey. Based on the preregistration, we then excluded 132 in the representative sample and 68 in the 60+ sample from all analyses for both failing a second attention check (which came between immediately prior to the eight accounts participants rated) and being in the bottom quartile of completion time, leaving a final *N*_rep_sample_ = 1,910 and *N*_60+_ = 937 (*N*_combined_ = 2,847).

#### Procedure

The survey was advertised as taking 20 min to complete, and participants were compensated $2.50 + 1 loyalty credit (valued at $0.67 within the Forthright Access participation system) upon completion. After providing informed consent, and prior to the experimental intervention, participants provided their party identification strength [American National Election Studies (ANES) measure], what social media platforms they used, and if they indicated Facebook and/or Twitter usages were asked typical hours of use on an average day in the past week on a 7-point Likert scale (“Did not use” to “More than 3 h per day”). Participants were then randomly assigned between-subjects to either play the video game *Snake* (the control condition), available via Google (https://www.google.com/fbx?fbx=snake_arcade), or to take the *Spot the Troll Quiz* (https://spotthetroll.org/), hosted on a separate website tab. Participants were informed they needed to spend a minimum of 7 min playing their respective game, at which point the arrow to progress would appear (a 7-min timer was visible to participants in the survey), and were asked to report the score they received in the game.

Postintervention, participants responded to the primary dependent variables. First, they responded to the 5-item social media inauthenticity self-efficacy scale on 1–7 Likert scales (“Strongly Disagree” to “Strongly Agree” with each statement, α = 0.84). The measure was inspired by Vraga and Tully (42). They then received instruction on and completed the eight-item novel troll-spotting task (order of profiles was randomized). Each profile contained the profile header and six tweets, and participants viewed each profile in isolation. Response options were “A Troll Account,” “A Real Account,” and “Not Sure” (always coded as incorrect). (See below for details on this novel troll-spotting task measure.) After rating all profiles, they were asked how many of the eight profiles they think they correctly identified. Participants then responded to the six-item affective polarization measure, where they rated on 0–100 thermometer scales their feelings toward Democratic/Republican voters, The Democratic/Republican party, and Trump/Biden voters. Participants then received instruction on, then rated their perceived reliability of the information in, four fake news headlines and two true news headlines drawn from Maertens et al. (19) on 1–7 Likert scales (“Very Unreliable” to “Very Reliable”). Participants then responded to basic demographic questions and were provided the opportunity to leave an open comment, and the study ended. Mean time to survey completion did not differ by condition (*P* = 0.786).

#### Analyses

Analyses were conducted using R statistical software version 4.2.2. All reported analyses utilized ordinary least squares (OLS) linear regression, and per the preregistration, all analyses controlled for political party identification (Democrat/Dem-Leaning, Republican/Rep-Leaning, and True Independent).

#### Development of the novel troll-spotting task

Of the eight profiles in the novel troll-spotting task, four were verified trolls who were active in June–August 2017 that Twitter subsequently banned as part of their efforts to combat coordinated information operations. Two accounts skewed liberal/Democratic, and two of the accounts skewed conservative/Republican. According to Twitter, these accounts were created by the Russian, Iranian, or Venezuelan governments. The other four accounts were real users who were directly recruited for the purpose of using their profiles in this measure, with the intent of collecting two real liberal and two real conservative accounts. For each account, participants saw the profile information/picture and six posts, just as in the *Spot the Troll Quiz*. We recruited candidates on Mechanical Turk via CloudResearch's toolkit (43) from November 2021 to January 2022. In order to participate, participants must have identified as either liberal or conservative, be age 22 or older, a user to Twitter, and a US citizen, in CloudResearch's prescreen. The purpose of the survey was overt to participants, namely, that we were recruiting them to ask permission to potentially use their likeness to create a scientific measure of individuals’ ability to discern authentic from inauthentic social media accounts. This survey took 1–2 min to complete and simply asked for individuals’ explicit permission to use their Twitter account as such and provide their Twitter handle if so. Participants were paid $0.60 upon completion, and pay was explicitly not contingent on providing permission. Upon collecting eight suitable accounts (four liberal and four conservative), images of the accounts were taken (or reconstructed from Twitter's data set of removed accounts) and lightly edited (e.g. removing advertisements from their feed and removing empty space), such that six tweets appeared below their profile. (See Supplementary material Section A for further details on the collection of the real accounts and pilot testing of the novel troll-spotting task.)

### Observational study

#### Data collection

Using the Twitter API on 2020 November 14, we collected all tweets that contained the exact text created by the “Share Your Score” link at the end of the *Spot the Troll Quiz*. We identified the author of each such tweet and collected and included the full public record of their Twitter output from 2020 August 15 to 2020 November 14. In the case of retweets, we also collected the author ID and account profile information of the retweeted accounts. On 2022 July 17, we attempted to recollect the account profile information for these same retweeted accounts and noted when those accounts were no longer present on the platform.

#### Empirical model

For each account, we limited the data set to 21 days before and after they shared their score and summed up a variety of outcomes by day, including the number of tweets, the number of retweets, the number of retweets of verified accounts (at time of tweeting), and the number of retweets from accounts that were deleted/suspended as of 2022 July 17.

For the outcome of account *i* on day *t* ($y_{it}$), we specify a panel event–study design in one of two forms. We include both a simple posttreatment dummy version of the form


(1) $$y_{it}=\;\alpha_{i}+\gamma_{t}+\beta^{*}\mathrm{pos}t_{it}+e_{it\;},$$

where β is the coefficient of interest, and a variable-impact version of the form


(2) $$y_{it}=\alpha_{i\;}+\gamma_{t\;}+\overset{}{\underset{j}{\sum }}\;\delta_{\;j\;}D_{ijt}+e_{it}$$

where $D_{ijt}$ is a dummy variable for each day after treatment (*j*), up to 5 days, and a final dummy for 6+ days, and the interest is in the coefficients $\;\delta_{1}\ldots \delta_{6}\;$. Both specifications also include account-specific fixed effect ($\alpha_{i}$) and day-specific fixed effects ($\gamma_{t}$). We estimated these models using the linearmodels pandas package (44), clustering standard errors by account.

## Supplementary Material

pgad094_Supplementary_Data

## Data Availability

All data from the experiment are publicly available at https://doi.org/10.17605/OSF.IO/KWMQV. The observational data from Twitter, by their very nature, include personally identifiable information as and such are not available publicly.
