# Supplementary material for: The Spot the Troll Quiz game increases accuracy in discerning between real and inauthentic social media accounts
Source: PNAS Nexus. 2023 Mar 22;2(4):pgad094. doi: 10.1093/pnasnexus/pgad094 (PMC10096901; doi:10.1093/pnasnexus/pgad094)
Supplement: pgad094_Supplementary_Data [file pgad094_supplementary_data.docx]

#### **Supplemental Materials**

##### **Table S1**

|  | *Polarization* | *Self-Efficacy* | *Accuracy Guess* | *Fake Reliability* | *Real Reliability* | *Total Accuracy* | *Troll Accuracy* | *LW Accuracy* | *RW Accuracy* | *Real Accuracy* | *Party ID Str* | *Age* |
| --- | --- | --- | --- | --- | --- | --- | --- | --- | --- | --- | --- | --- |
| *Polarization* |  |  |  |  |  |  |  |  |  |  |  |  |
| *Self-Efficacy* | -0.01 |  |  |  |  |  |  |  |  |  |  |  |
| *Accuracy Guess* | 0.02 | 0.33^***^ |  |  |  |  |  |  |  |  |  |  |
| *Fake Reliability* | -0.03 | 0.03 | -0.05^*^ |  |  |  |  |  |  |  |  |  |
| *Real Reliability* | 0.02 | 0.10^***^ | 0.11^***^ | 0.13^***^ |  |  |  |  |  |  |  |  |
| *Total Accuracy* | -0.02 | 0.12^***^ | 0.26^***^ | -0.15^***^ | 0.07^***^ |  |  |  |  |  |  |  |
| *Troll Accuracy* | -0.00 | 0.04^*^ | 0.22^***^ | -0.20^***^ | 0.01 | 0.76^***^ |  |  |  |  |  |  |
| *LW Accuracy* | -0.02 | 0.13^***^ | 0.15^***^ | -0.00 | 0.10^***^ | 0.71^***^ | 0.08^***^ |  |  |  |  |  |
| *RW Accuracy* | 0.00 | 0.09^***^ | 0.18^***^ | -0.13^***^ | 0.04^*^ | 0.76^***^ | 0.56^***^ | 0.56^***^ |  |  |  |  |
| *Real Accuracy* | -0.03 | 0.09^***^ | 0.21^***^ | -0.09^***^ | 0.07^***^ | 0.75^***^ | 0.59^***^ | 0.52^***^ | 0.15^***^ |  |  |  |
| *Party ID Str* | 0.37^***^ | -0.01 | 0.02 | 0.11^***^ | 0.01 | -0.02 | -0.02 | -0.01 | 0.01 | -0.04 |  |  |
| *Age* | 0.24^***^ | -0.23^***^ | -0.16^***^ | 0.02 | -0.04 | -0.18^***^ | -0.13^***^ | -0.13^***^ | -0.18^***^ | -0.09^***^ | 0.09^***^ |  |
| *Computed correlation used pearson-method with pairwise-deletion.* | | | | | | | | | | | | |

Note: These data do not include true independents. **P* < 0.05, ***P* < 0.01, ****P* < 0.001.

##### **Supplemental Section A**

***Further Information on the Development of Novel Troll Spotting Task*.** We initially collected 100 liberals and 100 conservatives, but ultimately collected an additional 100 conservatives in order to meet the criteria below. Of the 300 participants we collected, 195 provided permission to use their likeness. The research team then manually searched all 195 profiles for the following criteria: (1) the user must have a public profile, where they (2) posted/retweeted at least six posted between June 1st and August 31st, 2017 (the time frame when the Troll accounts were active), for which (3) at least one of the posts had clear political content aligned with theif self-reported ideology. Collecting 300 responses was necessary to obtain the two liberal and two conservative accounts that met these criteria. Throughout this process, the research team used their discretion and deep familiarity with the troll accounts these real accounts would be judged against to adjudicate the inclusion/exclusion of accounts based on these criteria.

Upon finding the eight suitable accounts, the novel accuracy measure was then piloted (N_final_ = 189) on Mechanical Turk via CloudPrime’s toolkit. Mean accuracy was 4.62 (SD = 1.32). Republican-leaning participants were less accurate on average than Democratic-leaning participants (β = -0.35, *t*(186) = -2.21 , *P* = 0.028), which motivated the decision to control for party identification in all analyses of the main study. However the results from the pilot met most other expectations. For four of the eight profiles there was no accuracy difference by party, for two Democrats were more accurate, and for two Republicans were more accurate. Accuracy was normally distributed in aggregate and widely varied across the accounts, only 6.8% of responses were “Not sure,” and accuracy was unrelated to demographic variables. Given these pilot results, we proceeded with the novel accuracy measure as constructed.

##### 
